# Supplementary material for: Microneurography as a minimally invasive method to assess target engagement during neuromodulation
Source: J Neural Eng. Author manuscript; Available in PMC 2024 Apr 13. (PMC10587909; doi:10.1088/1741-2552/acc35c)
Supplement: Verma 2023 Supplementary data [file NIHMS1929701-supplement-Verma_2023_Supplementary_data.pdf]

Supplementary Material 1 – Close-up photographs of recording electrodes

Supplementary Material 2 – Histology of great auricular nerve

Supplementary Material 3 – Great auricular nerve surgical pocket

Supplementary Material 4 – Facial nerve anatomy

Supplementary Material 5 – Great auricular nerve anatomy

Supplementary Material 6 – Non-functional electrodes

Supplementary Material 7 – Detrending of ECAPs from stimulation artifact

Supplementary Material 8 – Secondary outcomes in pre-registration

Supplementary Material 9 – Primary noise contribution in time series from low-frequency noise (e.g., cardiac related artifacts)

Supplementary Material 10 – Verifying authenticity of B-fiber recordings using signal propagation delay

Supplementary Material 11 – Data from all subjects

Supplementary Material 12 – Spontaneous activity recordings from cervical vagus nerve (cVN)

Supplementary Material 1 – Close-up photographs of recording electrodes

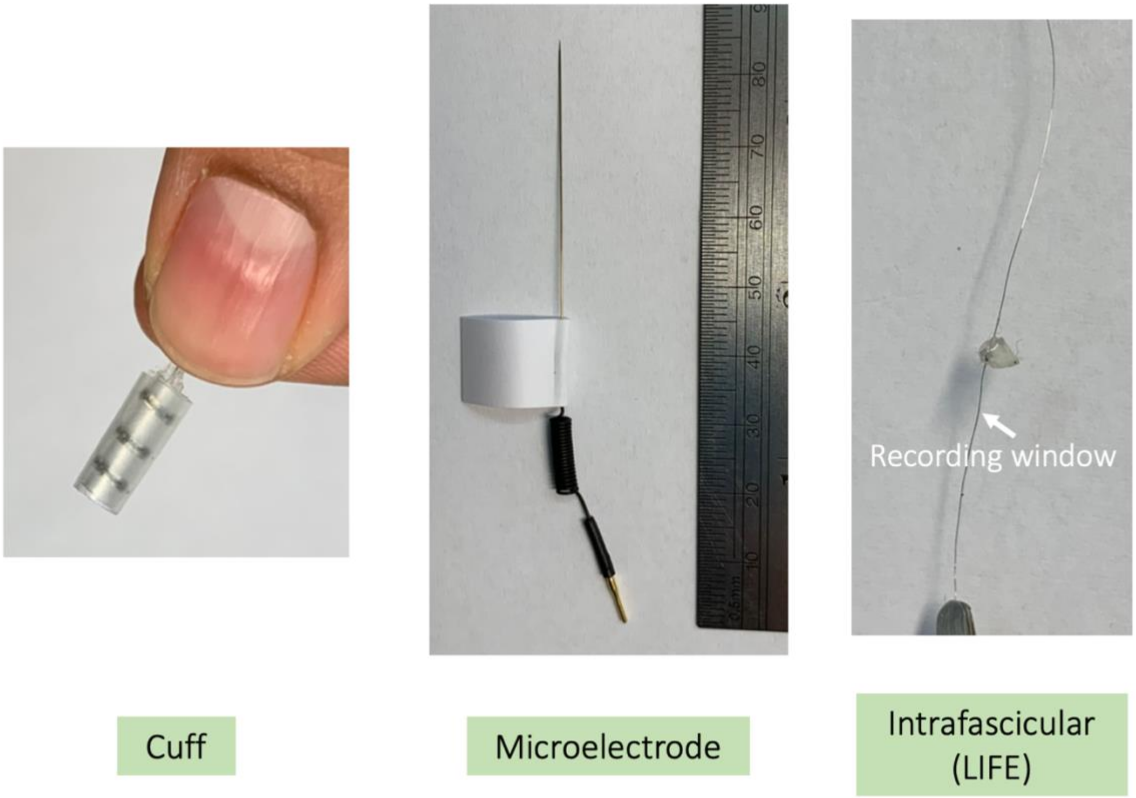

Figure S1-1: Close up photograph of three recording electrodes characterized in this study.

Supplementary Material 2 – Histology of great auricular nerve

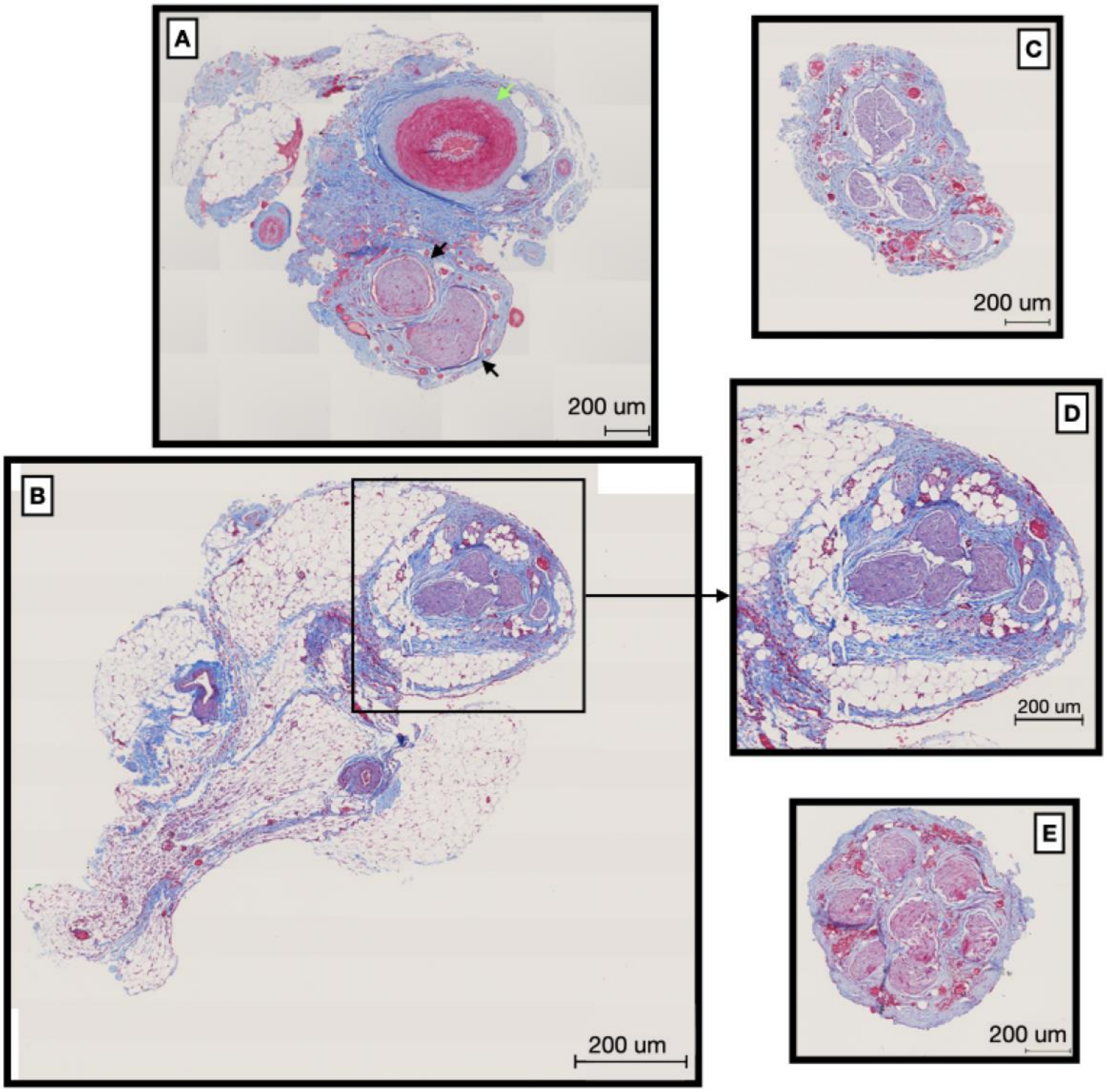

Figure S2-1: Representative histology of great auricular nerve cross sections (5 um) for four subjects. **(A)** Contains both the fascicles of the great auricular nerve (black arrow heads) and the associated artery (green arrowhead) **(D)** Zoomed region of **(B)**, showing fascicles of the great auricular nerve.

Supplementary Material 3 – Great auricular nerve surgical pocket

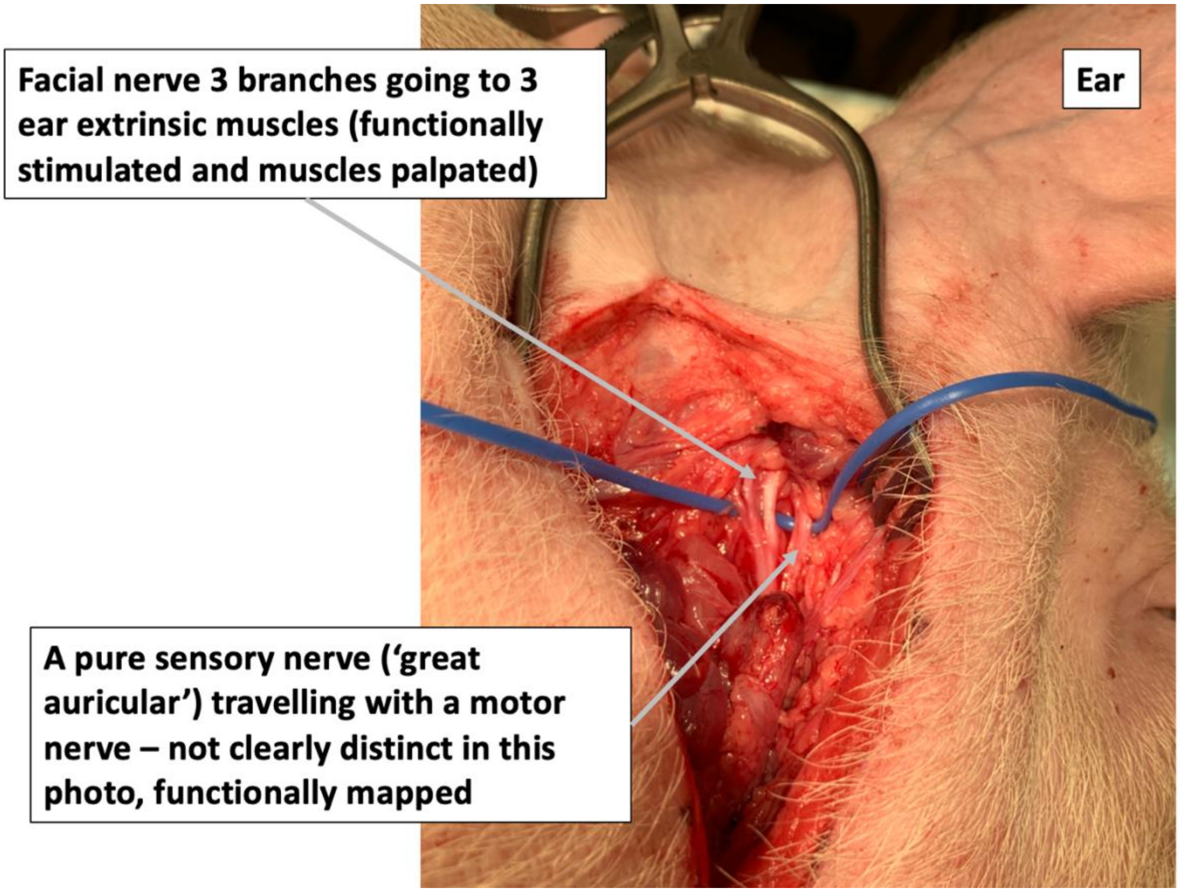

Figure S3-1: Surgical pocket to access great auricular nerve and surround anatomy. Functional mapping allowed differentiation between motor nerves (facial) and the sensory nerve (great auricular)

Supplementary Material 4 – Facial nerve anatomy

Facial nerve (pig):

1. caudal auricular nerve
2. internal auricular nerve
3. auriculopalpebral nerve
4. dorsal buccal branch
5. ventral buccal branch
6. stylohyoid branch
7. cervical branch

Note in human literature, 'anterior' and 'posterior' are used while in animal literature 'rostral' and 'caudal' are used respectively.

Caudal/posterior auricular nerve:
Initiates near stylomastoid foramen courses dorsal bifurcating into caudal and rostral branches. Both caudal and rostral branches course in association with caudal auricular artery and internal auricular nerve. Caudal branch is smaller of the two and innervates cervicoauricularis profundus muscle, with smaller branches innervating transverse and oblique auricular muscles as well as parotidoauricularis muscle. Innervates both intrinsic and extrinsic muscles of the outer ear.

Rostral/anterior auricular branch:
Continues a dorsal course and passes between retroauricular fat pad and ventral surface of the cervicoauricularis profundus muscle. In this area the rostral branch gives off smaller branches to styloauricularis muscle. These branches penetrate the auricular cartilage at helicine fissure and innervate the helicine muscle, terminating in the styloauricularis m.

Internal auricular branch:
Originates from dorsal margin of facial nerve and courses dorsal.
Divides into two to three branches before entering auricular cartilage and ramifying the skin of the inner surface of the auricular cartilage.

Auriculopalpebral nerve:
Courses dorsal communicating with auriculotemporal nerve then dividing into rostral auricular branches which innervate the auriculares rostrales muscles and a zygomatic branch to the frontoscutularis, levator anguliocauli medialis and orbicularis oculi muscles.

*Sisson and Grossman's "The Anatomy of the Domestic Animals", 1975.

Supplementary Material 5 – Great auricular nerve anatomy

The great auricular nerve (GAN) courses dorsally after emerging from the sternomastoid muscle. Traveling below the platysma muscle and coursing in association with the external jugular vein to the parotid margin. At this point the GAN begins to divide into its terminal branches. The GAN bifurcates into two primary branches with each of the primary branches dividing further.

The first division is the anterior branch. This branch courses rostrally over the parotid gland bifurcating into superficial and deep branches. The superficial branch innervates the skin over the parotid gland on the face. The deep branch penetrates the SMAS and parotid fascia and communicates with the facial nerve within the parotid gland (Yang et.al JPRAS 2015).

The posterior branch continues dorsal towards the posterior base of the ear and in association with the lateral auricular vein. It continues coursing around the posterior margin of the ear branching out to innervate the skin over the mastoid process. Another branch enters the cartilage at the base of the ear and supplies the anterior lower third of the ear. In humans this branch has been identified and referred to as the lobular branch (Sharma et al 2016). In pigs the lobular or lower third of the ear does not have a fatty lobule, the lobular equivalent of the pig would include the exterior skin around the tragus, antitragus, and intertragic notch areas of the ear. The posterior branch then continues along the posterior/caudal margin of the ear to innervate the posterior surface/skin of the auricle.

Some studies have suggested that this “lobular” branch be identified as a separate branch that supplies sensory innervation to the lower third of the ear. There are several studies that suggest that the GAN branches have four to five main configurations, we however did not dissect the entire configuration from each pig but did note that locations for the nerve configurations varied between animals (Sharma et al ASPS 2016; Lefkowitz et al., Aesthetic Surgery Journal, 2013; Yang et al. JPRAS 2015).

We found very little information on peripheral nerve anatomy or function in the pig model. Furthermore, the information can be misleading and inconsistent compared with surgical observations. We did not conduct a complete anatomical study for our purposes. However, future studies are warranted if the GAN in pig is to become a standard model.

Supplementary Material 6 – Non-functional electrodes

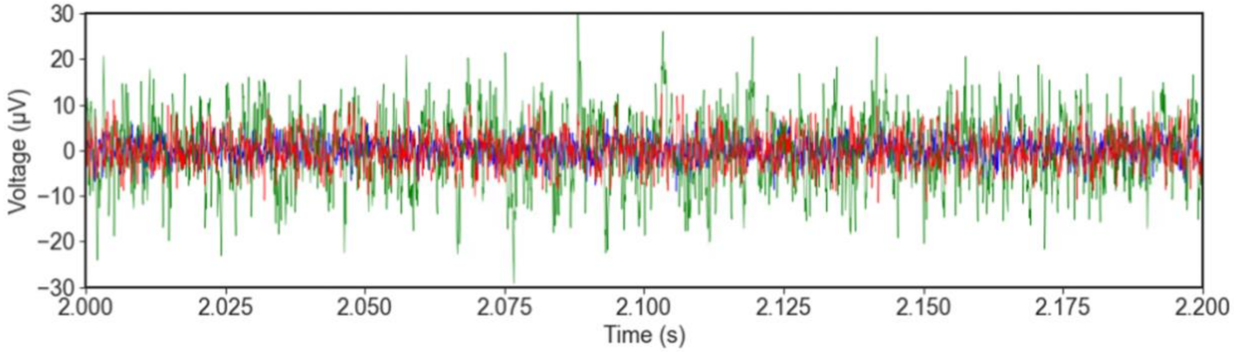

Figure S6-1: Subject 1 vagus micro 2 was a non-functional electrode classified by the high noise floor (green trace) compared to the blue and red traces (figure below) and no ECAP signal even at 5 mA of stimulation

Subject 1 great auricular micro 2 was a non-functional electrode classified by the high noise floor (figure in Supplementary Material 11 – Data from all subjects).

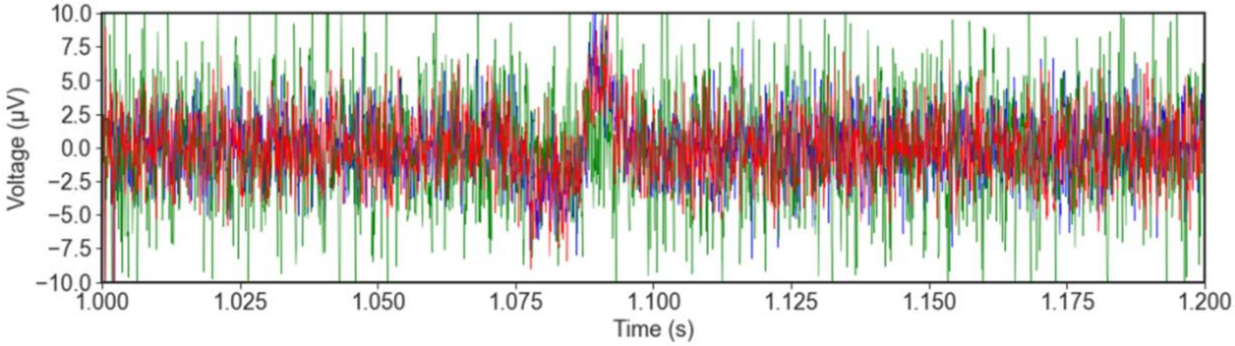

Figure S6-2: Subject 4 vagus cuff 2 was a non-functional electrode classified by the high noise floor (green trace) compared to the blue and red traces (figure below) and noted as physically disconnected at the end of the recording.

Supplementary Material 7 – Detrending of ECAPs from stimulation artifact

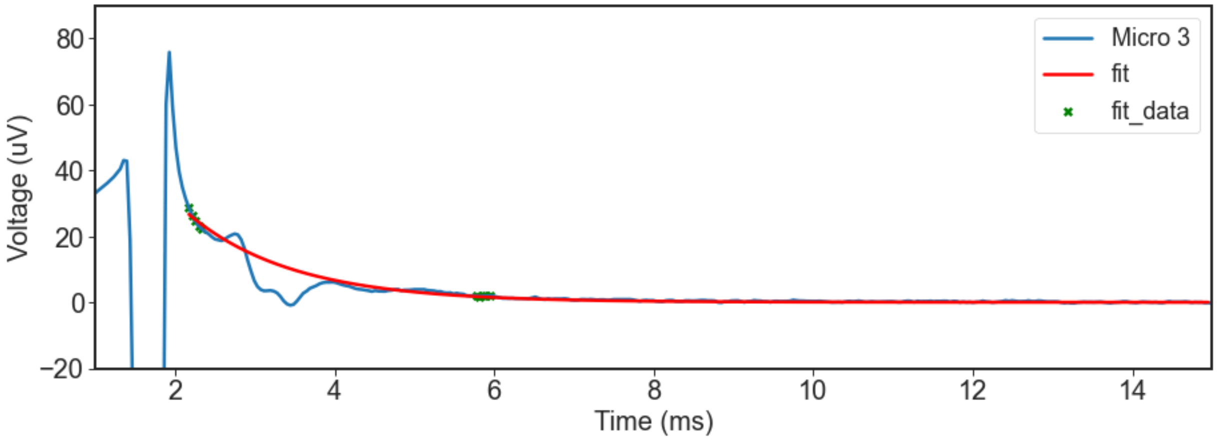

Figure S7-1: Only ECAPs from Subject 6 microelectrode recordings during the vagus dose-response amplitude sweep were contaminated by stimulation artifact. This is a representative fit of an exponential decay to the stimulation artifact in the ECAP trace.

Supplementary Material 8 – Secondary outcomes in pre-registration

Sequential analysis, total $\alpha = 0.05$ propagated from primary analysis):

- EC10 point on vagus Aβ-fibers ECAP AUC dose-response plot
  - ANOVA between 3 recording electrode types to show EC10 point is different with design of recording electrode. A logistic growth function will be used to fit the dose-response plot and calculate the EC10 point. The EC10 point is a proxy for the stimulation level at which the ECAP is first detectable.
- Comparison of baseline spontaneous activity recorded on cervical vagus nerve
  - Micro > cuff (one-sided t-test)
- EC10 point on vagus B-fibers ECAP AUC dose-response plot
  - ANOVA between 3 recording electrode type to show EC10 point is different with design of recording electrode.
- ECAP AUC of cervical vagus B-fibers (first distinguishable ECAP in 3-15 m/s conduction speed – allow 20% tolerance on conduction speed) at 5 mA of stimulation. Per animal, all ECAPs will be normalized to maximum B-fiber ECAP recorded at 5 mA stimulation.
  - Cuff > LIFE (one-sided t-test)
  - Cuff > micro (one-sided t-test)
- Comparison of non-invasive evoked (by TES at skin area of stroking response) neural signals on great auricular nerve comparing microelectrode (n = 6) with cuff (n = 3) and LIFE (n = 3)
  - Micro > cuff (one-sided t-test)
  - Micro > LIFE (one-sided t-test)
- Cervical vagus Aβ-fibers ECAP AUC dose-response plot transition region location and width consistency across animals for recording electrode type
  - Hypothesis: Cuff has least variability across animals while microelectrode is most sensitive due to variable placement relative to active neural source
  - Statistical test: Compare variability in fit parameters of (n=6) animals across 3 different electrode types
  - ANOVA of fit parameters without follow-up
- Tripolar vs. bipolar ECAP AUC amplitude ECAP Aβ- and B-fibers
  - Tripolar > bipolar (one-sided t-test)

Supplementary Material 9 – Primary noise contribution in time series from low-frequency noise (e.g., cardiac related artifacts)

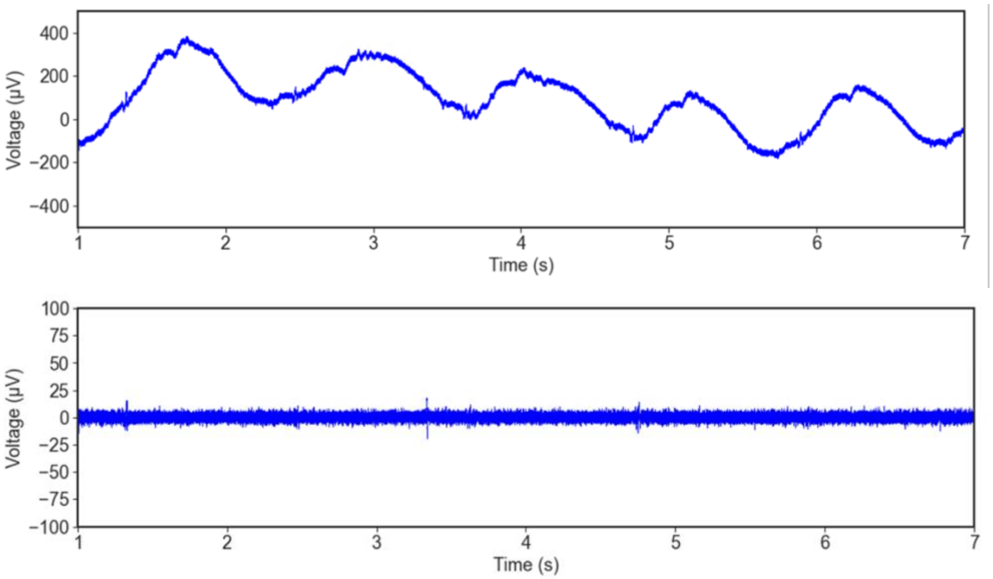

Figure S9-1: Microelectrode traces from subject 1 before (top) and after (bottom) filtering. High RMS noise in unfiltered microelectrode time series is coming largely from low frequencies.

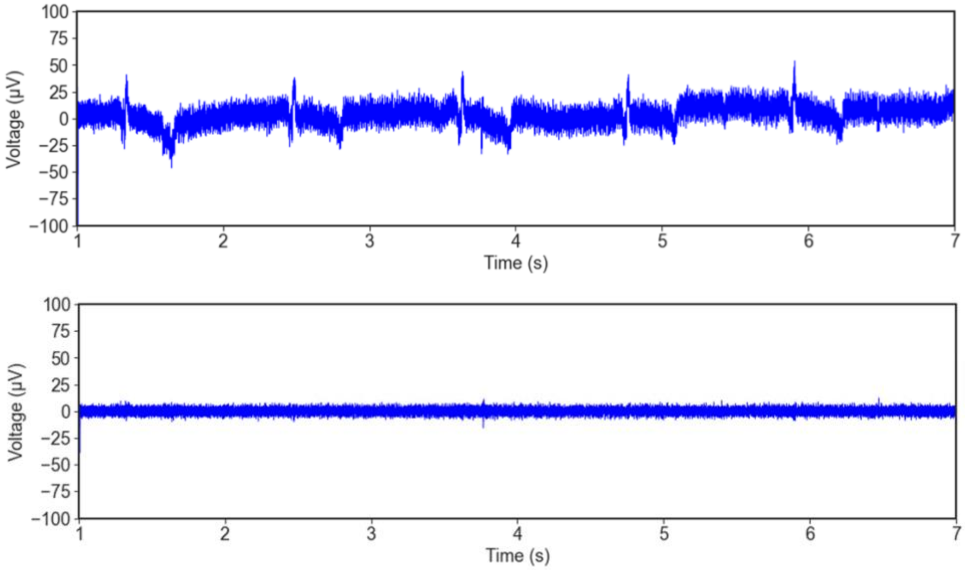

Figure S9-2: (top) An example of cardiac related artifact appearing in electrophysiology recordings, which may be mistaken for a neural signal. The cardiac related effect could be cardioballistic in nature or from the electrical activity of the heart, depending on the setup of the reference and recording electrode. (bottom) Removed by high pass filtering (fc = 100 Hz). See Fig. 5B in main paper for additional artifact examples. Cuff traces from subject 1.

Supplementary Material 10 – Verifying authenticity of B-fiber recordings using signal propagation delay

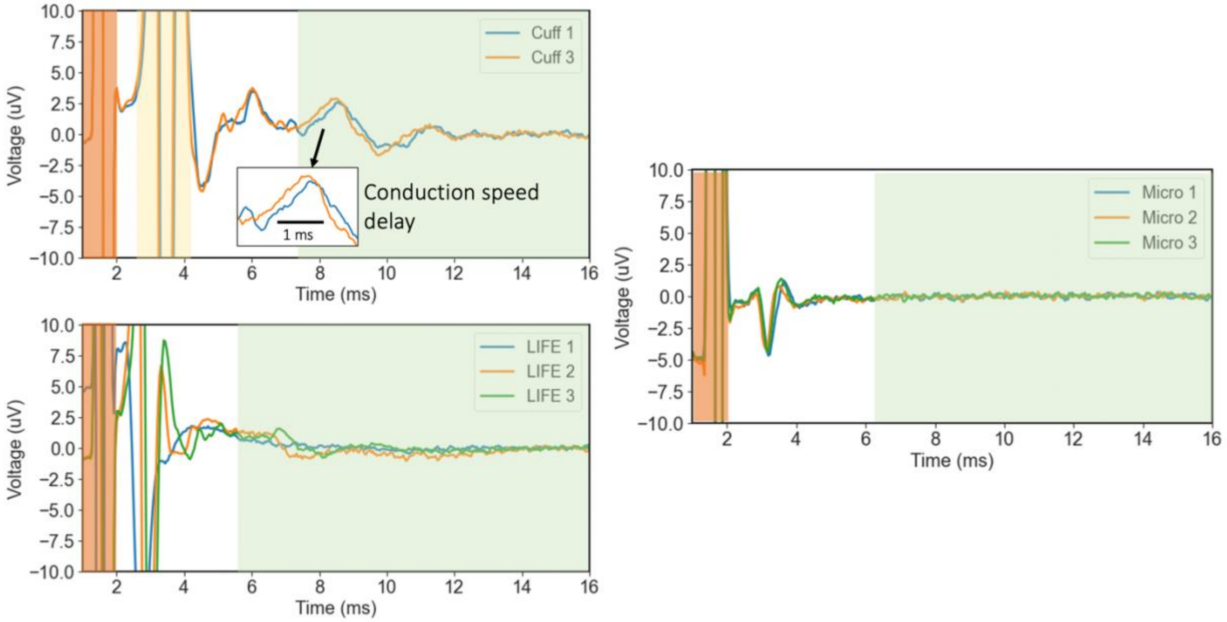

Figure S10-1: ECAP during 10 mA of stimulation on the vagus nerve in subject 4. (top left) with recording cuff (bottom left) with intrafascicular electrodes and (right) with microneurography microelectrode. Stimulation artifact shaded in orange, Aβ-fiber ECAP shaded in yellow, and B-fiber ECAP region shaded in green. Authenticity of B-fiber ECAP is suggested by signal propagation delay in cuff and intrafascicular electrode recording but not in microneurography microelectrode recording.

Supplementary Material 11 – Data from all subjects

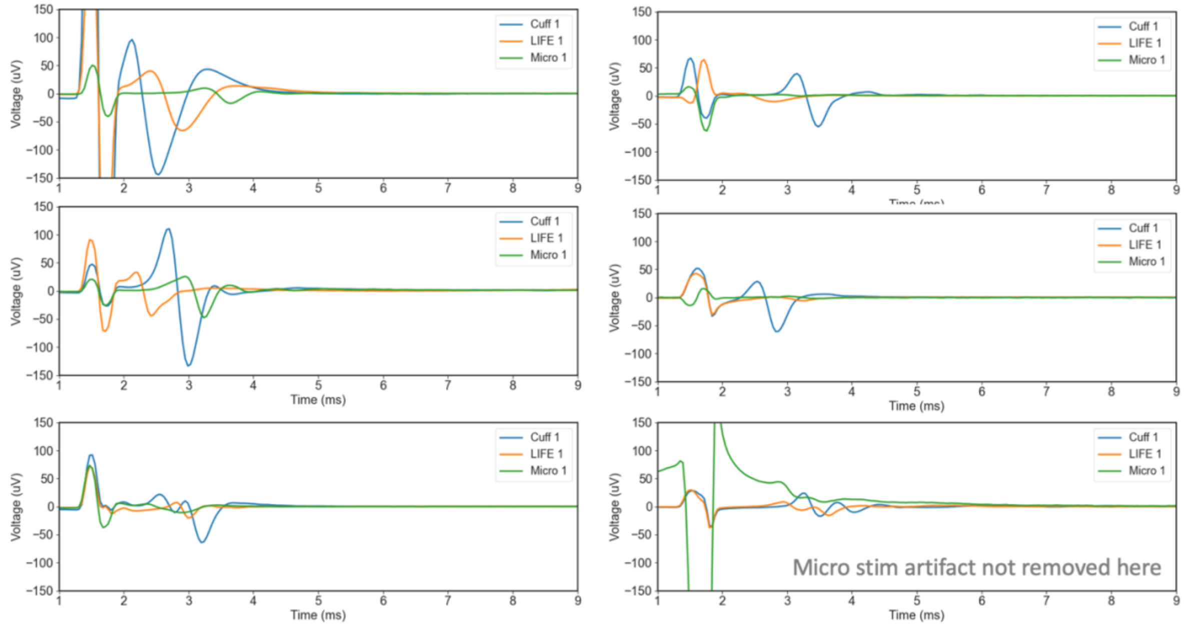

Figure S11-1: Aβ ECAP at 1.5 mA of stimulation on cervical vagus nerve from all subjects. (top-left) subject 1 to (bottom-left) subject 3 and (top-right) subject 4 to (bottom-right) subject 6. Cuff consistently records the largest ECAP. Apparent triphasic morphology in subject 1 cuff may be due to stimulation artifact recovery.

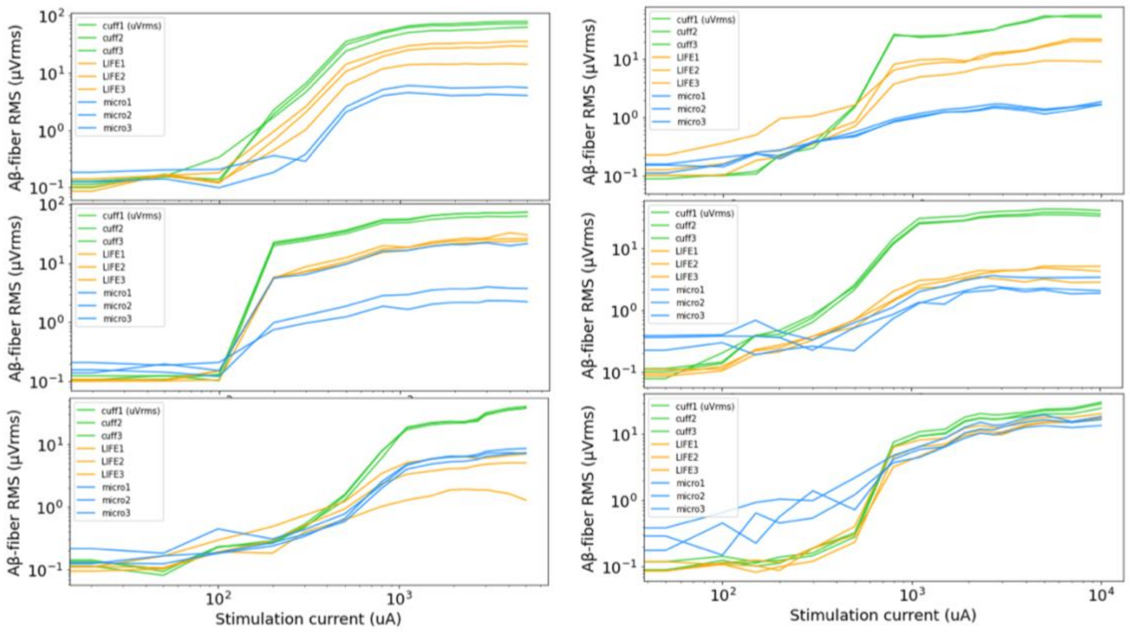

Figure S11-2: Aβ-fiber dose-response curves during vagus nerve stimulation from all subjects. (top-left) subject 1 to (bottom-left) subject 3 and (top-right) subject 4 to (bottom-right) subject 6.

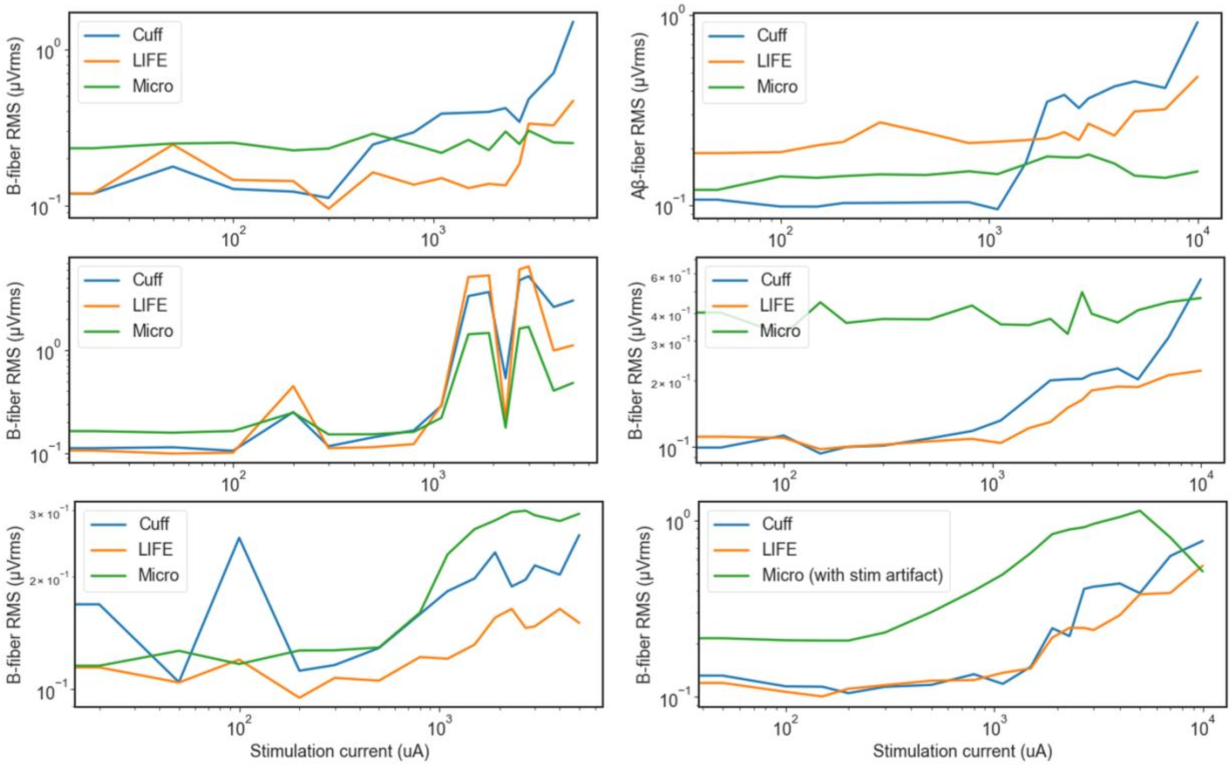

Figure S11-3: B-fiber dose-response curves during vagus nerve stimulation from all subjects. (top-left) subject 1 to (bottom-left) subject 3 and (top-right) subject 4 to (bottom-right) subject 6. An insufficient dose of Vecuronium (muscle blocker) was used in subjects 1-3 so EMG contamination of the B-fiber dose-response curves is likely. Not subject 6 microneurography microelectrode recordings are contaminated by stimulation artifact.

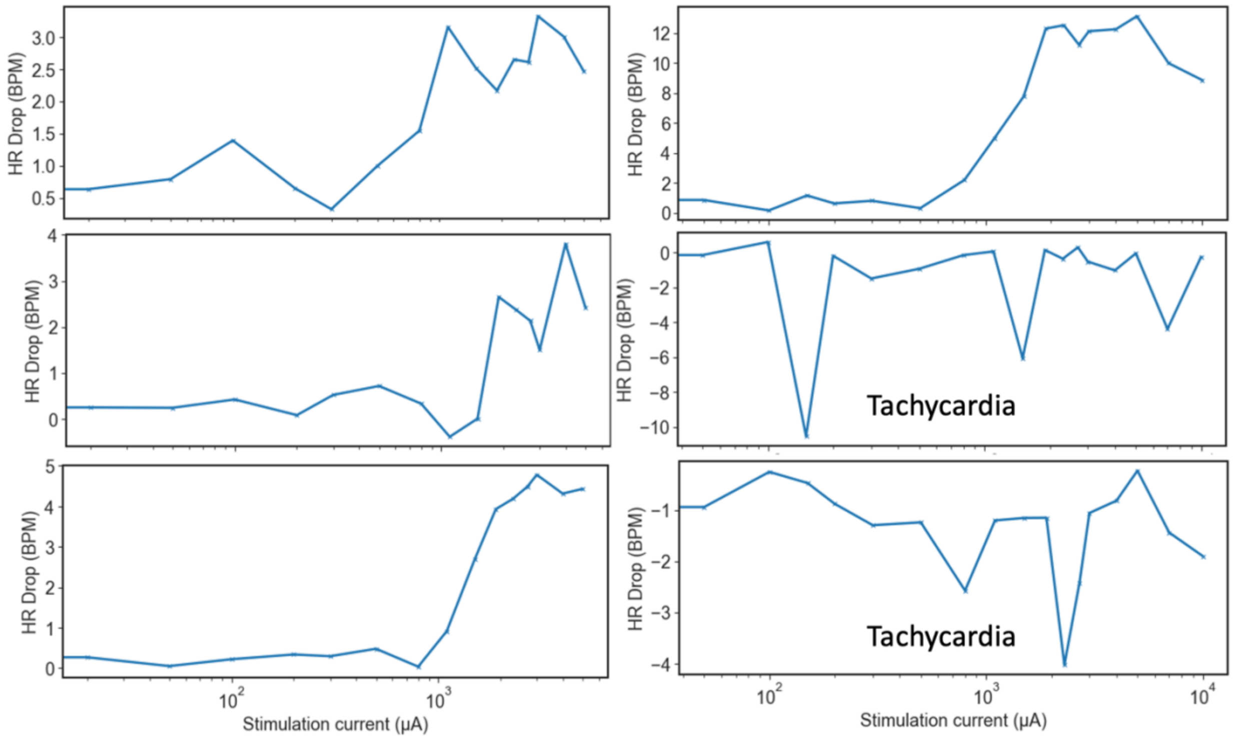

Figure S11-4: Stimulated evoked heart rate change dose-response curves during vagus nerve stimulation (VNS) from all subjects. (top-left) subject 1 to (bottom-left) subject 3 and (top-right) subject 4 to (bottom-right) subject 6. Subjects 5 and 6 are showing a tachycardia response instead of the canonical bradycardia response to VNS. Note that subjects 1-3 had the vagus experiment performed in the afternoon and were under greater doses of anesthesia compared to subjects 4-6 that had the vagus experiment performed in the morning and were under lower doses of anesthesia.

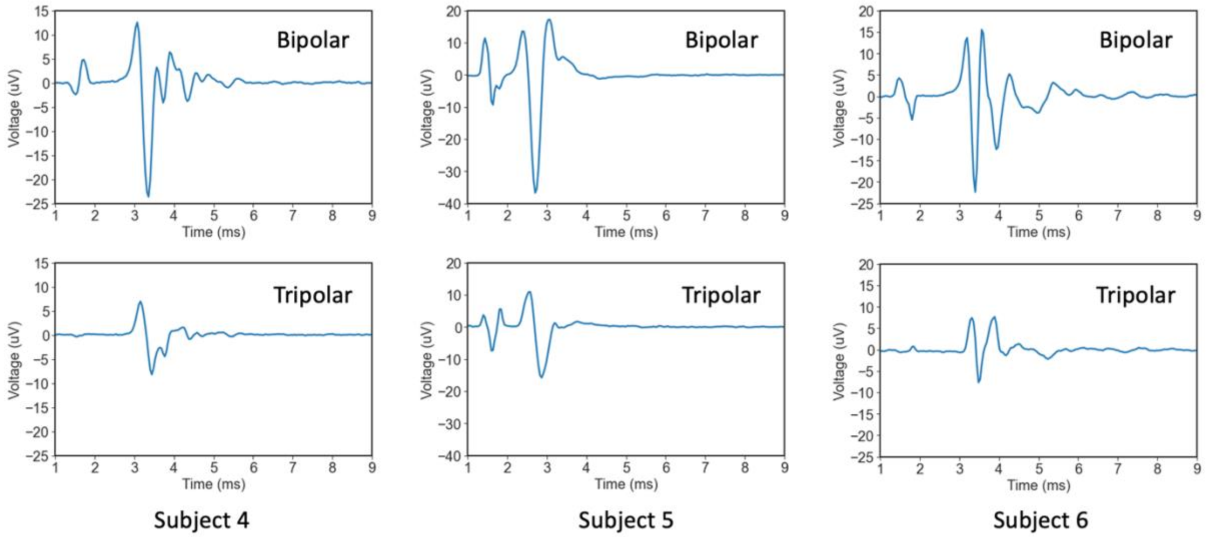

Figure S11-5: Tripolar vs. bipolar reference cuff recordings from subjects 4 (left), 5 (center), and 6 (right). Stimulation artifact is consistently smaller in tripolar. ECAP magnitude is consistently smaller in tripolar by factor of ~2.2x compared to bipolar reference.

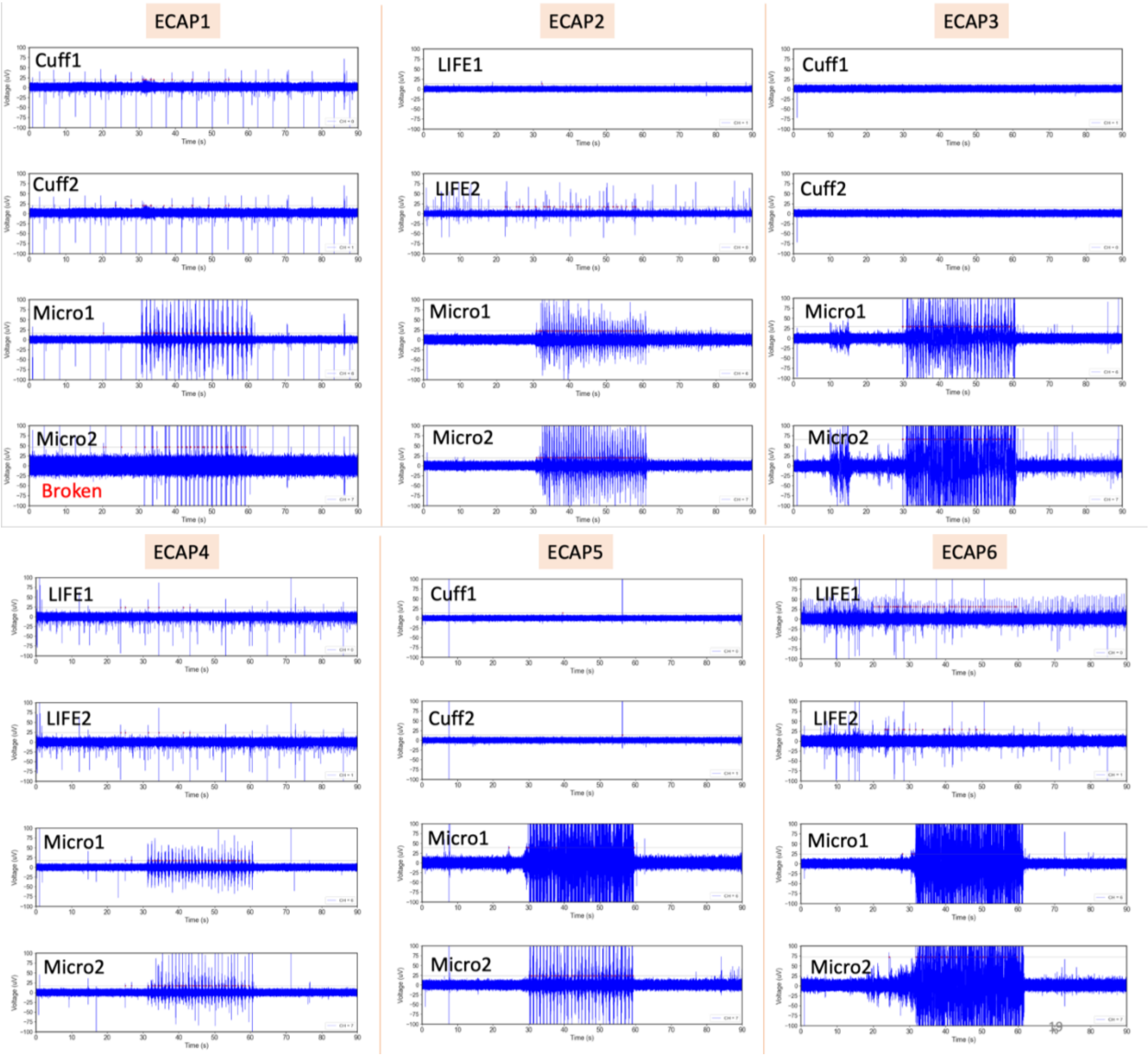

Figure S11-6: Spike count from sensory stroking evoked naturally occurring activity on all subjects. (top-left) subject 1 to (bottom-right) subject 6. 90 seconds of recording shown: first 30 seconds are quiescent with no stroking, 30-60s are on-target stroking at the region of the auricle innervated by the great auricular nerve, and 60-90s are off-target stroking at a region of the auricle not innervated by the great auricular nerve.

Supplementary Material 12 – Spontaneous activity recordings from cervical vagus nerve (cVN)

None of the electrode recordings showed clear and repeatable spontaneously occurring neural activity in the cVN. Signals that initially appeared neural were likely motion artifacts as they persisted more than 20 minutes after the nerve was transected cranial and caudal to the recording electrodes. Further, the artifacts were more pronounced in subjects 1-3, where the subjects experienced more tremor as insufficient muscle paralytic, vecuronium, was administered, compared to subjects 4-6.

Representative recordings from 1 cuff contact (channel 0), 1 LIFE electrode (channel 5), and 1 microneurography electrode (channel 8) per subject:

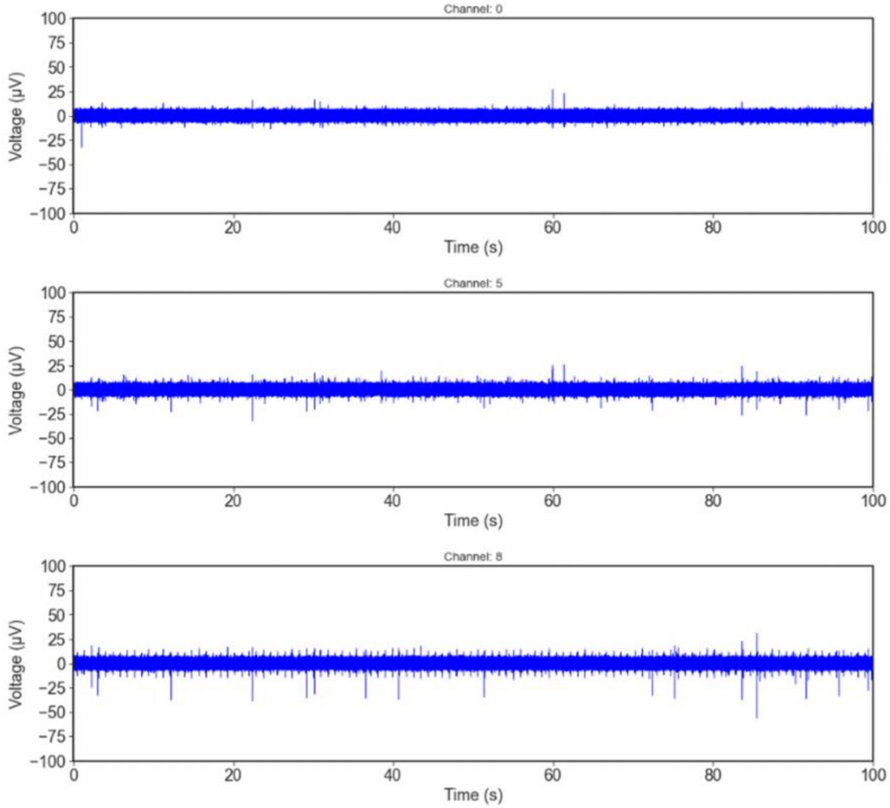

Figure S12-1: Subject 1 cVN spontaneous activity recording.

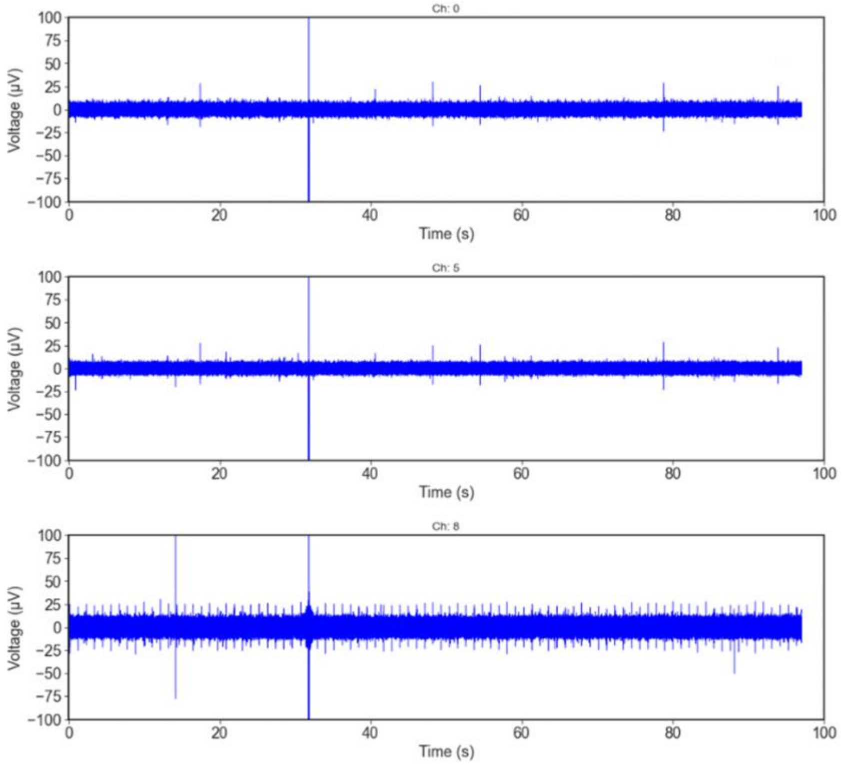

Figure S12-2: Subject 2 cVN spontaneous activity recording.

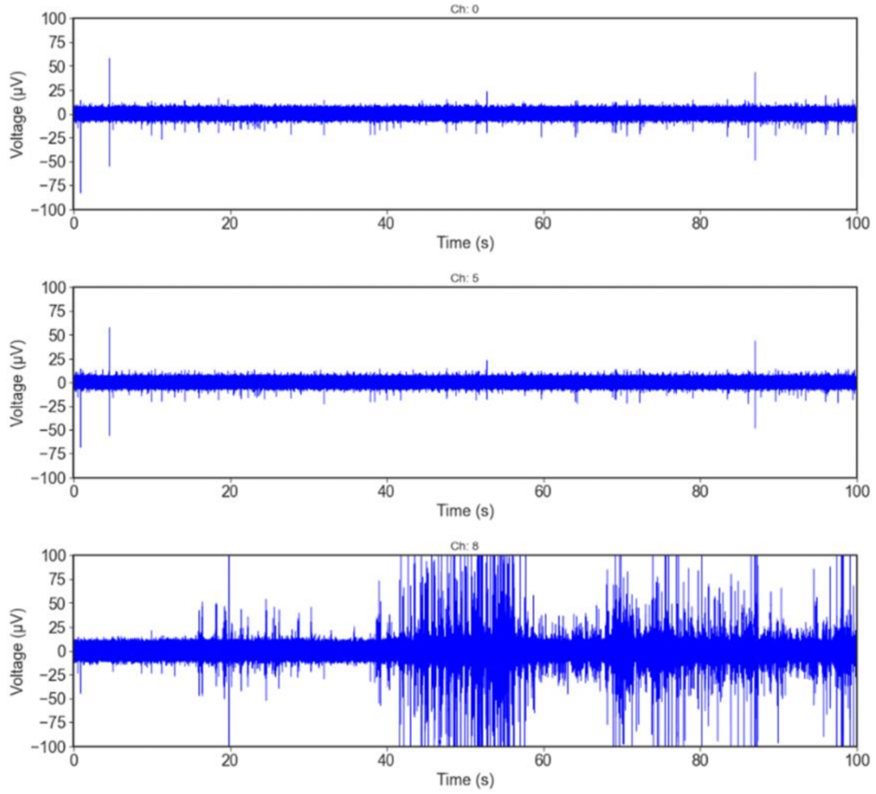

Figure S12-3: Subject 3 cVN spontaneous activity recording.

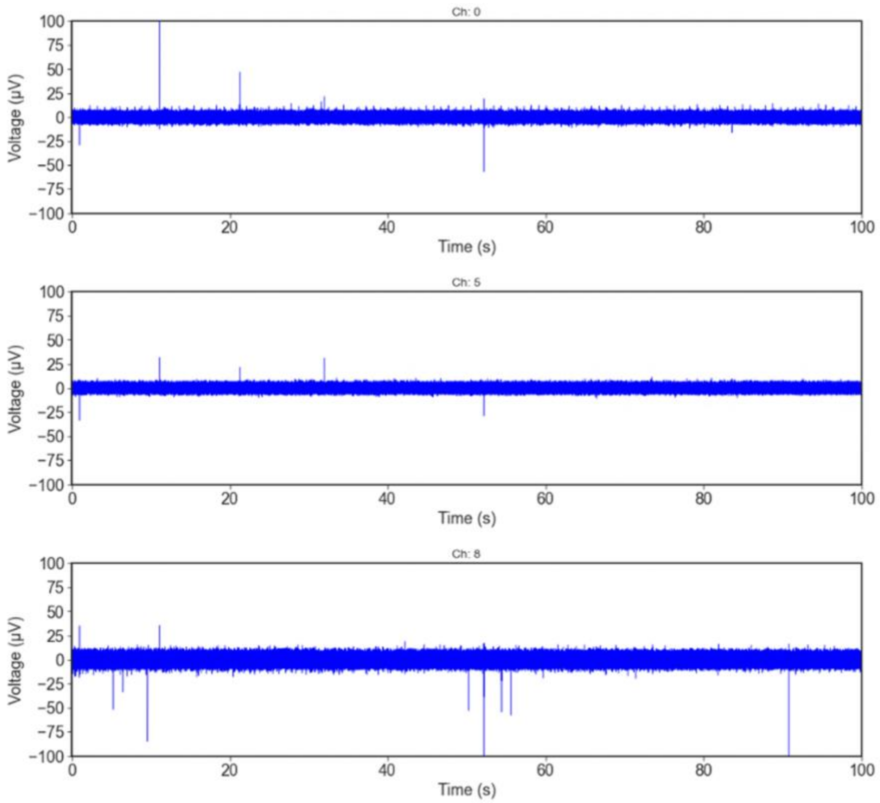

Figure S12-4: Subject 4 cVN spontaneous activity recording.

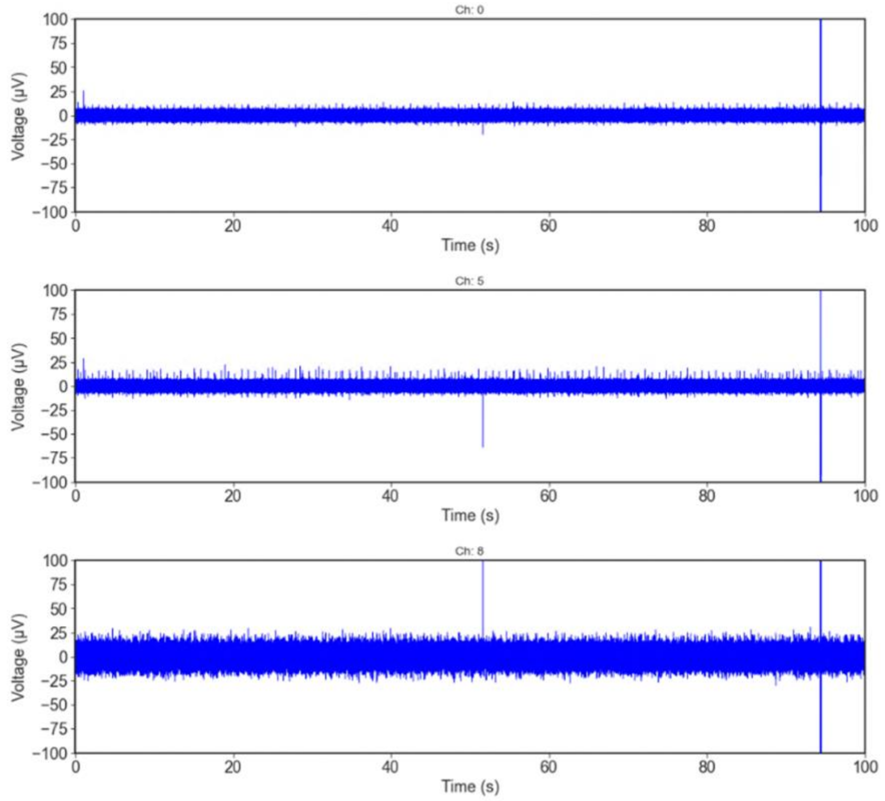

Figure S12-5: Subject 5 cVN spontaneous activity recording.

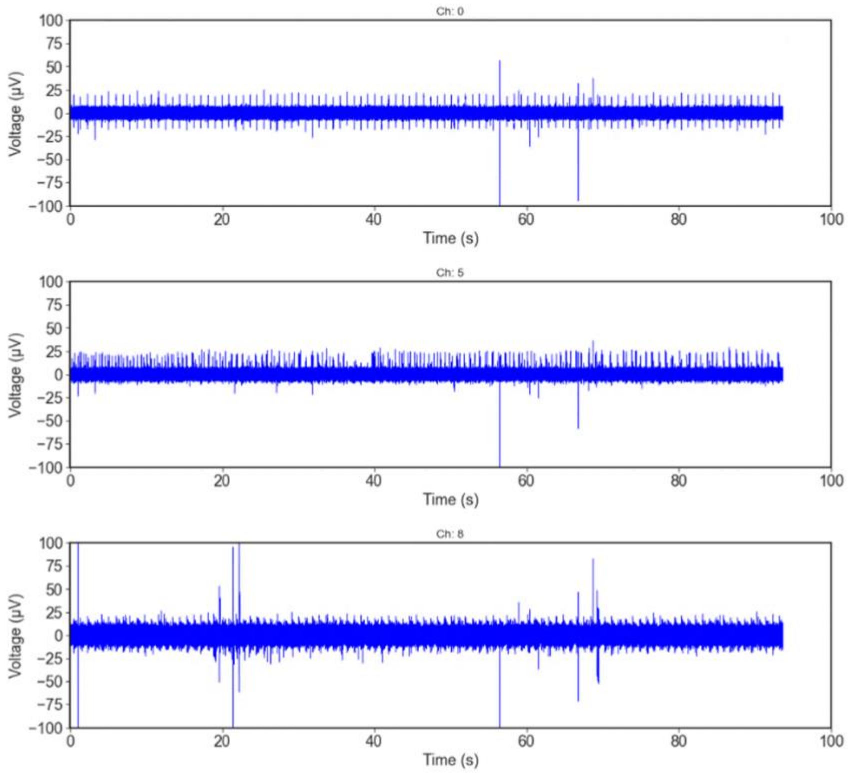

Figure S12-6: Subject 6 cVN spontaneous activity recording.
